# A systematic review of measures of HIV/AIDS stigma in paediatric HIV-infected and HIV-affected populations

**DOI:** 10.7448/IAS.19.1.21204

**Published:** 2016-10-06

**Authors:** Carole Ian McAteer, Nhan-Ai Thi Truong, Josephine Aluoch, Andrew Roland Deathe, Winstone M Nyandiko, Irene Marete, Rachel Christine Vreeman

**Affiliations:** 1 Department of Pediatrics, Indiana University School of Medicine, Indianapolis, IN, USA; 2 Academic Model Providing Access to Healthcare (AMPATH), Eldoret, Kenya; 3 Department of Child Health and Paediatrics, School of Medicine, College of Health Sciences, Moi University, Eldoret, Kenya

**Keywords:** HIV stigma, paediatric, HIV stigma measures, quantitative tools, HIV-affected, HIV-infected

## Abstract

**Introduction:**

HIV-related stigma impacts the quality of life and care management of HIV-infected and HIV-affected individuals, but how we measure stigma and its impact on children and adolescents has less often been described.

**Methods:**

We conducted a systematic review of studies that measured HIV-related stigma with a quantitative tool in paediatric HIV-infected and HIV-affected populations.

**Results and discussion:**

Varying measures have been used to assess stigma in paediatric populations, with most studies utilizing the full or variant form of the HIV Stigma Scale that has been validated in adult populations and utilized with paediatric populations in Africa, Asia and the United States. Other common measures included the Perceived Public Stigma Against Children Affected by HIV, primarily utilized and validated in China. Few studies implored item validation techniques with the population of interest, although scales were used in a different cultural context from the origin of the scale.

**Conclusions:**

Many stigma measures have been used to assess HIV stigma in paediatric populations, globally, but few have implored methods for cultural adaptation and content validity.

## Introduction

In 2013, there were an estimated 3.2 million children under the age of 15 years living with HIV, with over 90% living in sub-Saharan Africa [1]. Families describe HIV stigma as a major barrier to access and adherence to HIV care for children, but relatively little is known about the experiences of HIV stigma among families with HIV-infected children and adolescents or how stigma is related to their physical, psychological and social outcomes [2–6]. For example, stunted growth and delayed bodily development with perinatal HIV infection may be sources of stress and anxiety for adolescents and lead to social isolation [7]. Families also report that the fear of stigma prevents them from taking important transitional steps such as disclosing a child's HIV status to the child, as they worry about subsequent stigma [8]. The impact of HIV stigma likely varies over a child's development from childhood into adolescence and may impact family members in the same household in different ways. HIV stigma may also impact HIV-affected individuals [9]. For example, children orphaned by parental HIV infection may be affected by HIV stigma and discrimination [10].

The objective of this review is to examine the construction and utilization of HIV stigma measures in paediatric HIV-infected and HIV-affected populations, globally. Reliable and valid measures of HIV-related stigma and its closely related constructs are needed to track the impact of interventions targeting stigma reduction. To date, there are relatively few data on how to measure HIV stigma among paediatric populations and in resource-limited settings. To address this gap, we sought to identify and describe quantitative HIV stigma measures used in paediatric populations that are HIV-infected or HIV-affected, with critical examination of the creation of scales or measurement tools for use within paediatric populations.

## Methods

To conduct this systematic review, we searched online databases including MEDLINE, EMBASE, PsycINFO, Academic Search, ERIC, CINAHL, Social Work Abstracts, Scopus and Web of Science as of 15 January 2015. The search strategy was (“social stigma,” “stereotyping,” “prejudice,” “discrimination,” “social perception,” “shame,” “social marginalization,” “social isolation,” “social distance,” “social exclusion,” “fear,” “self concept” OR “self perception”) AND (“human immunodeficiency virus,” “Acquired Immunodeficiency Virus,” “Acquired Immunodeficiency Syndrome” OR “HIV Infections”) AND (“paediatrics,” “child” OR “adolescent”). The search was supplemented by truncated keywords (e.g. stereotyp*) and bibliography review.

Because the systematic review aimed to explore stigma measures previously utilized for children, in addition to measure development, we did not include specific search terms for measurement tools (e.g. “measure” and “questionnaire”). Inclusion criteria were the following: (1) administering a quantitative HIV/AIDS-related stigma measurement tool, (2) study population including HIV-infected or HIV-affected children or adolescents =18 years of age, (3) peer-reviewed publication and (4) published in English language. Since many studies explored stigma with paediatric populations and their caregivers, we only included studies that utilized a stigma tool with the paediatric sub-population. As this was a youth-focused review, we excluded studies that technically fit inclusion criteria by including individuals <18 years of age, but which had fewer than five participants under age 18 because their target participants were not children, adolescents or young adults. We included studies from all geographic locations. Articles that reported findings from the same study population, using the same measurement tools over the same period of time were counted as a single study, for which we combined the results of their multiple publications into a single report.

Two authors (NTT and CIM) independently reviewed all articles and determined whether the studies met the inclusion criteria. Disagreements were discussed with a third reviewer (RCV) to reach consensus. Data regarding study location, target population, stigma measures used and any correlates of stigma measured were extracted from the included studies.

To assess the quality of the HIV/AIDS stigma measures, a quality criteria tool by Terwee [11] was utilized. Content validity was assessed based on the extent to which the domain of interest was comprehensively sampled by the questionnaire items. Internal consistency assessed the extent to which items on a scale were inter-correlated. Construct validity was scored as the extent to which scores on a questionnaire relate to measures in a manner consistent with a derived hypothesis. Each criterion was scored by two authors (NTT and CIM) with a (+) for meeting the criterion, (?) for doubtful design, (−) for studies that reported the criterion but did not meet the appropriate threshold and (0) was scored for no information provided. From studies meeting inclusion criteria, the revised quality criteria tool was applied to 22 of the studies.

## Results and discussion

**Figure 1:**
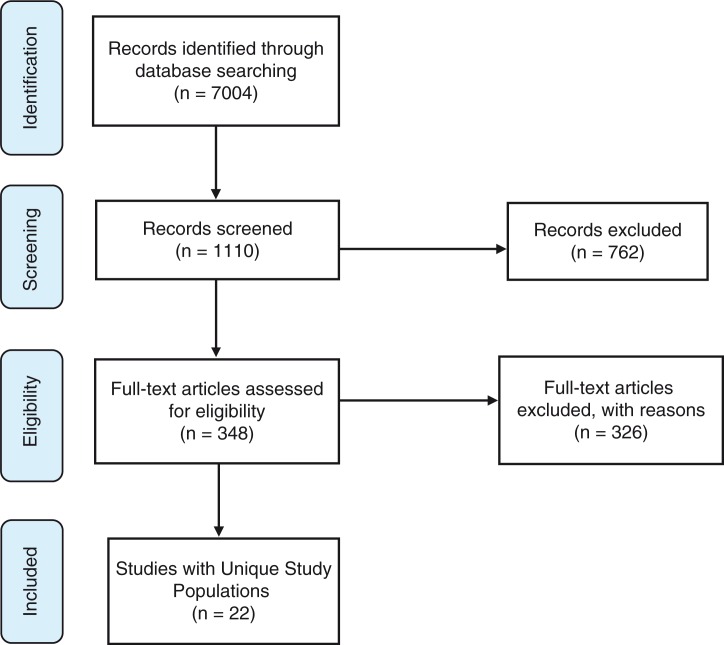
PRISMA database search results [12].

The search terms identified 7004 titles, which were reviewed to determine whether they met the inclusion criteria. Following the title review, 1110 abstracts were reviewed, and then 348 full articles were reviewed. Twenty-seven articles met the inclusion criteria (Figure 1).

Of these 27 articles, the compilation of the multiple publications describing the same study populations resulted in 22 unique studies to include in the final analysis [10,13–18]. Some studies with the same population used different stigma measures and were therefore considered separate studies in this review (see Table 1).

**Table 1:** Study locations and populations and stigma measures used

Author and year	Location	Population	Stigma measure	Correlates explored
Boyes *et al*. (2013)	South Africa	HIV affected (*N*=723)	Brief HIV Stigma by Association Scale (α=0.78–0.87)	Stigma by groups
Chi *et al*. (2014)	China	aHIV affected (*N*=1625)	SACAA (α=0.84–0.88)	Depression
Clum *et al*. (2009)	United States	HIV infected (*N*=147)	HIV Stigma Scale Subscales (α=0.90)	Depression
Cluver *et al*. (2008)	South Africa	aHIV affected (*N*=1025)	Modified HIV Stigma Scale (α=0.88)	Stigma by groups
Cluver. *et al*. (2013)	South Africa	HIV affected (*N*=6002)	Brief HIV Stigma by Association Scale (α=0.87)	Stigma by groups
Dowshen (2009)	United States	HIV infected (*N*=42)	HIV Stigma Scale (α=0.79–0.94)	Depression
Fair (2008)	United States	HIV affected (*N*=10)	Modified HIV Stigma Scale	Stigma
Fongkaew (2014)	Thailand	HIV infected (*N*=30)	HIV Stigma Scale (α=0.95)	Adherence
Lin X *et al*. (2010)	China	aHIV affected (*N*=1625)	Perceived Stigma Scales (α=0.86, 0.87)	Stigma by group
Mason *et al*. (2010)	United States	HIV affected (*N*=27)	Brief Stigma by Association Scale (α=0.86)	Stigma
Mavhu *et al*. (2013)	Zimbabwe	HIV infected (*N*=10)	New questions	
Murphy *et al*. (2006)	United States	HIV affected (*N*=118)	Modified HIV Stigma Scale (α=0.80 and 0.68)	Delinquency
Onuoha & Munakata (2010)	South Africa and Uganda	HIV affected (*N*=952)	Detroit Measure of Discrimination (α=0.78)	Social discrimination
Radcliffe *et al*. (2010)	United States	HIV infected (*N*=40)	Swendeman's Scale	Sexual risk behaviours
Rongkavilit *et al*. (2010)	Thailand	HIV infected (*N*=70)	Thai Youth HIV Stigma Scale (α=0.96)	Mental health, HrQoL
Swendeman *et al*. (2006)	United States	HIV infected (*N*=147)	Modified Enacted and Perceived Scales (α=0.53–0.83)	Social rejection
Wang (2012)	China	aHIV affected (*N*=1221)	SACAA, Perceived and Enacted Stigma Scales (α=0.88, 0.88)	Trauma, depression
Wright *et al*. (2007)	United States	HIV infected (*N*=48)	Brief Measure of HIV Stigma (α=0.72–0.88)	Stigma
Wiklander *et al*. (2013)	Sweden	HIV infected (*N*=58)	HIV Stigma Scale for children-8 (α=0.78–0.81)	HrQoL
Zhao *et al*. (2012)	China	aHIV affected (*N*=1625)	SACAA, Enacted and Perceived Scales (α=0.88)	Mental health
Zhao *et al*. (2010)	China	aHIV affected (*N*=1625)	SACAA, Perceived Stigma Scale (α=0.86)	Mental health
Zhao *et al*. (2011)	China	HIV affected (*N*=1625)	New questions (α=0.87)	AIDS knowledge

a Represents studies with the same population studied, but with different stigma scales or subscales used.

### Populations

Of the 22 final articles, eight studies were conducted in the United States [9,16,19–24], five in Africa [10,15,25–27], eight in Asia [28–35] and one in Sweden [36]. Seven unique studies had large sample sizes (*N*>900): four studies with the same orphaned, vulnerable children and comparison children were conducted in China [31–34], and three were conducted in Africa [10,15,25]. Five of the studies focused on HIV-affected children [10,25,27,31,37], nine focused on HIV-infected children [16,19,21,23,24,26,28,29,36] and eight focused on comparing groups of HIV/AIDS orphans, orphans from other illnesses and vulnerable children [9,20,22,30,32–35].

### HIV-infected population and stigma scales

Nine of the studies focused on implementing or validating a stigma scale with HIV-infected paediatric or young adult participants [16,19,21,23,24,26,28,29,36]. Four of these studies focused on special populations: two recruited only participants who were young men who have sex with men (MSM) [19,24], one recruited only behaviourally infected female participants [26] and one recruited only substance-using young people [23]. The five remaining studies, all of which were smaller studies (*N*<900), included general populations of HIV-infected youth [16,21,28,29,36].

### HIV-affected population and stigma scales

Seven unique studies administered stigma measures to HIV-affected children [9,15,20,22,27,30,35]. Two studies, Fair *et al*. [22] and Murphy *et al*. [20], measured HIV stigma in children of HIV-infected mothers.

The remaining studies assessed between-group differences among HIV-affected children (HIV/AIDS-orphaned children and vulnerable children) and children not affected by HIV (children orphaned by other causes) [14,15,27,33,34]. Boyes and Cluver [15] and Cluver *et al*. [10,25] found that HIV-affected youth in South Africa had higher stigma scores than non-affected youth with the Brief Stigma by Association Scale. Onuoha and Munakata [27] used the modified Detroit Area Study Measure of Discrimination as a measurement of HIV stigma to assess social discrimination of children orphaned by AIDS in South Africa and Uganda and found that HIV/AIDS-orphaned children reported higher levels of discrimination and higher levels of psychological distress than the comparison groups. Zhao *et al*. [35] explored HIV/AIDS-related knowledge and HIV stigma in comparison groups of children orphaned by HIV/AIDS, children living with HIV-infected parents and comparison children that were non-HIV affected and found that within the children in the HIV-affected groups, those children with higher HIV/AIDS knowledge had lower stigma scores. In China, Zhao *et al*. [34] found that, in a comparison study with HIV/AIDS orphans and vulnerable children, the Stigma Against Children Affected by HIV (SACAA) measure had good construct validity and was positively associated with psychopathological symptoms and negatively associated with well-being in both HIV-affected children and comparison children.

## Stigma scales

Many different HIV stigma scales were used in paediatric populations. Some studies utilized existing measures, but with modifications (see Table 2) and others developed their own questions to measure stigma.

**Table 2:** Development of new stigma scales

Author and year	Original scale used	New scale items
Boyes (2013)	HIV Stigma by Association Scale for Adolescents	Brief Stigma by Association Scale
Chi (2014)	New	Enacted Stigma Scale
Cluver (2008)	HIV Stigma Scale	4-item Modified HIV Stigma Scale
Mason (2010)	HIV Stigma Scale	HIV Stigma by Association Scale for Adolescents
Mavhu (2013)	New	Questionnaire with Stigma Domains
Rongklavilit (2010)	HIV Stigma Scale	Thai Youth HIV Stigma Scale
Swendeman (2006)	Perceived Stigma and Enacted Stigma (Sowell 1997)	Shortened Perceived Stigma and Enacted Stigma
Wikilander (2013)	HIV Stigma Scale	HSSC-8
Wright (2007)	HIV Stigma Scale	Brief Measure of Stigma (HSS-B)
Zhao (2010)	New	SACAA
Zhao (2011)	New	Personal Stigma Toward People Living with HIV/AIDS

### The HIV Stigma Scale

The HIV Stigma Scale (HSS-B) developed by Berger *et al*. [38] was the most frequently utilized and modified assessment tool. Developed from two rounds of content review and validated in a large, diverse sample of HIV+ adults in the United States, the scale consists of 40 items divided into four subscales: personalized stigma, disclosure concerns, negative self-image and concern with public attitudes toward people with HIV. Each item is measured on a 4-point scale. Dowshen *et al*. [19] utilized the full HSS-B for their study involving HIV+ MSM young adults in United States. Confirmatory factor analysis was bypassed, but Cronbach's alphas were provided for total scale score (0.94) as well as disclosure concerns (0.79), personalized stigma (0.93), negative self-image (0.84) and public attitudes (0.91) subscales. To assess HIV stigma in HIV-infected adolescent females in the United States, Clum *et al*. [16] used the HSS with the negative self-image and disclosure subscales with a Cronbach's alpha of 0.90.

In Sweden, Wiklander *et al*. [36] shortened and modified the HSS for HIV+ Swedish youth. The authors utilized a “think-aloud” methodology with the target population, factor analysis of items from the HSS and qualitative review by an expert panel. The subsequent 8-item scale, entitled HIV Sigma Scale for Children, HSSC-8, included only two items for the disclosure subscale, and the personalized stigma subscale was removed entirely [36]. Murphy *et al*. [20] utilized 19 items from the HSS after conducting confirmatory factor analysis on the original HSS subscale items. Fair *et al*. [22] and Murphy *et al*. [20] modified the questions to ask if the children perceived stigma because their mothers had HIV, but were not otherwise altered for the young, sero-negative target population [39].

Mason *et al*. [9] created a 23-item HIV Stigma by Association Scale for Adolescents from content analysis with the target population, cognitive interviews and assessments of content validity of the full HSS items to reflect stigma for children of HIV-infected mothers. Boyes *et al*. [15] further modified Mason *et al*.'s [9] scale for South African HIV-affected youth through qualitative interviews and item selection, resulting in a validated 10-item Brief Stigma by Association Scale, which was further examined in South African HIV-affected populations with Cluver *et al*. [25].

Wright *et al*. [21] administered the full HSS to 48 HIV-infected participants and modified the measure to create a Brief Measure of Stigma for HIV-positive youth, a 10-item scale called the Brief Stigma Scale. Overall, the Brief Stigma Scale showed good internal consistency and validity. In South Africa, Cluver *et al*. [10] created a 4-item stigma scale based on Wright *et al*.'s [21] Brief HSS and adapted the scale for non-infected orphans using qualitative interviews with the target population, literature review and expert input.

In Thailand, Rongkavilit *et al*. [29] and Fongkaew *et al*. [28] used translated versions of the full HSS to assess stigma in Thai youth living with HIV. Rongkavilit *et al*. [29] utilized the full HSS on Thai youth living with HIV/AIDS and created an abbreviated 12-item scale through factor analysis and found no overall differences between the new scale and the HSS for the population studied. Fongkaew *et al*. [28] utilized a mixed methods approach for assessing HIV stigma in the Thai youth population and found that qualitative findings corroborated the stigma scores, particularly in the personalized stigma subscale.

### The Perceived Public Stigma Against Children Affected by HIV Scale

One stigma scale was used in China with additional stigma subscales by five studies in HIV-affected populations [30–34]. Zhao *et al*. [34] created the Perceived Public Stigma Against Children Affected by HIV/AIDS (SACAA), which measures children's perceptions of public stigma against affected children. The SACAA was developed based on a literature review of stigma measures, qualitative fieldwork with HIV-affected participants in China and investigator input. The SACAA consists of 10 items, measured on a 4-point scale, with subscales on social exclusion, purposive avoidance and perception of being inferior. Chi *et al*. [30] administered the SACAA to HIV-affected children in China along with a 12-item Enacted Stigma Scale; Wang *et al*. [32] utilized the SACAA in addition to a 14-item Enacted Stigma Scale; and Lin *et al*. [31] utilized the SACAA and a 10-item Personal Stigma Scale with positive ratings for content validity and internal consistency. Zhao *et al*. [33] compared the SACAA with three other stigma scales: Perceived Public Stigma Against People Living with HIV/AIDS (PLWHA) (Cronbach's alpha=0.86), Personal Stigmatizing Attitudes Against PLWHA (Cronbach's alpha=0.87) and Enacted Stigma scales (Cronbach's alpha=0.88). Zhao *et al*. [33] found that the different stigma measures captured different psychosocial outcomes of interest, and thus, each scale was slightly different in the type of stigma being measured.

### Other stigma scales

In the United States, Swendeman *et al*. [23] modified a pre-existing scale for use with substance-using HIV-infected young women. Based on literature review and focus groups with HIV+ women, Sowell *et al*. [40] developed the HSS-S. The HSS-S was modified by Swendeman *et al*. [23] and resulted in 11 enacted stigma item and 7 perceived stigma items. Although no mention of target population involvement was made, factor analyses were performed and Cronbach's alphas provided. Two enacted stigma questions were excluded due to low analysis values, and the remaining divided into avoidance, abuse and social rejection subscales (alphas=0.71, 0.59 and 0.53), while the perceived stigma items were divided into avoidance, social rejection and shame subscales (alphas=0.83, 0.67 and 0.69). The seven perceived items were utilized by Radcliffe *et al*. [24] to assess stigma among young HIV+ MSM in the United States. The response format was altered to yes/no, but items were not otherwise altered, and no factor analyses or Cronbach's alphas were reported.

In Africa, Mavhu *et al*. [26] used a mixed methods approach to assess stigma of HIV+ Africaid support group attendees. The quantitative measure was developed from questionnaires previously validated in Zimbabwe as well as newly developed and pretested questions. Factor analysis and Cronbach's alpha were not mentioned for this measure of stigma. Onuoha and Munakata [27] used the modified Detroit Area Study Measure of Discrimination to assess social discrimination of children orphaned by AIDS in South Africa and Uganda. Although the target population was not involved in item selection, the measure was administered to a focus group of adolescent children in both countries to assess cultural validity in young children, and the Cronbach's alpha for the scale was 0.78.

In China, Zhao *et al*. [35] developed new questions to assess children's attitudes toward PLWHA. The 10 questions were developed from a literature review on HIV stigma with children affected by HIV/AIDS and had a Cronbach's alpha of 0.87.

## Cultural adaptations of stigma scales

For quantitative tools that measure psychosocial constructs such as HIV stigma, common research procedures suggest that researchers assess the cultural relevance and validity of the tool if it is being used in a cultural context different from where the tool originated. Many studies in this review adapted pre-existing HIV stigma measures to refine items for use with paediatric populations or target populations [9,20–22]; however, only three studies revised HIV stigma tools within the cultural context of interest, using the target population as panel experts or in qualitative inquiry to address cultural relevance of stigma items [15,34,36] Fongkaew *et al*. [28] used a pre-existing Thai translation of the HSS that had previously been used with a Thai adult population and Rongkavilit *et al*. [29] conducted a factor analysis of the Thai translated scale, which eventually resulted in the Thai Youth HSS. Mavhu *et al*. [26] utilized qualitative inquiry to help guide question construction for the stigma items asked of HIV-infected children in Zimbabwe. The study does not discuss any further factor analysis conducted for the items constructed with the target population.

## Quality assessment

Assessed quality varied greatly across the studies (see Table 3). The studies designed to create and validate measures of stigma varied significantly in their quality, which may carry implications for the validity of the scales they created. On the quality criteria rating scale, only the scales developed by Boyes *et al*. [15] and Zhao *et al*. [34] received positive ratings for both content validity and internal consistency. Although Mason *et al*. [9] and Wiklander *et al*. [36] received positive ratings for content validity, they received indeterminate ratings for internal consistency due to their small sample sizes. Rongkavilit *et al*. [29] and Wright *et al*. [21] similarly received indeterminate internal consistency ratings due to small sample sizes and also received negative ratings for content validity, as they did not adapt items for age or culture.

**Table 3:** Quality criteria checklist scoring

Quality criteria	(+) Meeting criteria	(−) No information provided	(?) Doubtful design	(0) Reported criteria but did not meet threshold
Content validity	Cluver (2008)	Dowshen (2009)	Mavhu (2013)	
	Wiklander (2013)	Fongkaew (2014)	Lin (2010)	
	Mason (2010)	Fair (2008)	Zhao (2012)	
	Boyes (2013)	Murphy (2006)	Zhao (2011)	
	Zhao (2010)	Rongkalivit (2010)	Chi (2014)	
	Onuoha (2010)	Wright (2007)	Clum (2009)	
		Radcliffe (2010)		
		Swendenman (2006)		
		Cluver (2013)		
Internal consistency	Dowshen (2009)	Mavhu (2013)	Fongkaew (2014)	
	Murphy (2006)		Fair (2008)	
	Cluver (2008)		Rongkalivit (2010)	
	Boyes (2013)		Wright (2007)	
	Cluver (2013)		Wiklander (2013)	
	Swendenman (2006)		Mason (2010)	
	Lin (2010)		Onuoha (2010)	
	Zhao (2012)		Radcliffe (2010)	
	Zhao (2010)		Zhao (2011)	
	Chi (2014)		Clum (2009)	
Construct validity	Wright (2007)	Mason (2010)	Fongkaew (2014)	Dowshen (2009)
	Wiklander (2013)		Fair (2008)	Murphy (2006)
	Boyes (2013)		Rongkavilit (2010)	
	Radcliffe (2010)		Cluver (2008)	
	Swendeman (2006)		Cluver (2013)	
	Lin (2010)		Onuoha (2010)	
	Zhao (2012)		Mavhu (2013)	
	Zhao (2010)		Zhao (2011)	
	Chi (2014)		Clum (2009)	

## Discussion

This systematic review sought to explore HIV stigma in paediatric HIV-infected and HIV-affected populations and how it can best be measured. Many studies utilized existing stigma measures and modified the measures to better assess the construct of stigma within their population of interest (e.g. HIV-infected children, HIV-affected children and children orphaned by HIV/AIDS). Based on the results of the systematic review, we found that, among the relatively small group of measures developed for measuring HIV stigma, very few have been validated for children or used in resource-limited settings. Those that have been developed and validated with children were primarily used with HIV-affected, rather than HIV-infected, children.

The HSS by Berger *et al*. [38], which has been extensively tested and validated in adult populations, was the most frequently utilized measure in the paediatric populations reviewed. Using measures previously validated among adults may not directly relate to HIV-infected or HIV-affected children. Children may be exposed to different social environments or different forms of stigma than HIV-infected adults and therefore may not experience or report discrimination in the same manner as adults. In addition, it is difficult to know how well the item construction or vocabulary is understood by children as this has seldom been examined. Therefore, future studies with paediatric HIV-exposed or HIV-infected populations should aim to formulate developmentally appropriate HIV stigma questions to best measure the impact of HIV stigma on the paediatric population. Qualitative investigation, including cognitive interviewing to assess the understanding, comprehension, and recall of children related to these questionnaire items would be useful.

Across the literature on HIV stigma, the number of studies that utilized a quantitative measure of paediatric HIV stigma was extremely limited. Of those studies, the majority included only small participant sample sizes of less than 200. There were no large-scale studies (with *N*>200) done in the United States, Europe or South America, nor were there any large studies focusing on children living with, rather than affected by, HIV/AIDS. The difference in experiences and effects of HIV stigma among paediatric HIV-affected and HIV-infected children remains unknown. It is plausible that the effects of experiencing HIV stigma are more harmful to the psychosocial development and well-being of HIV-infected children than for HIV-affected children.

Moreover, the long-term impact of stigma on HIV-infected children as they become adolescents and adults has not been studied among the large populations of children currently growing up with HIV in resource-limited settings. As HIV-infected adolescents are the only group of PLWHA among whom the death rate continues to increase, it is critical that we evaluate factors shaping the lives of these youth [41]. Adolescence is a developmental stage in which the opinions and perceived judgments of peers hold particularly strong weight. Future research should also explore the impact of stigma at the various developmental stages of children, adolescents and adults. The relationship of HIV stigma and medication adherence also yielded mixed results, and the measures themselves also varied [26,28]. With the subjectivity of qualitative inquiry, the qualitative measures of addressing adherence may not have accurately captured the association of stigma and adherence. The clinical impact of stigma on children and adolescents remains to be explored.

The majority of the studies in this review were conducted in the United States, Asia and Africa. In a review of HIV/AIDS Stigma by Mahajan *et al*. [42], the authors called for validation of stigma measures in diverse settings. We found that tools that were modified from pre-existing tools and conducted in a similar cultural context had consistent results with psychosocial constructs [10,15,25]. This suggests that, even with the modifications made, the overall tool had consistency in measuring the same construct of stigma. Some of the studies that utilized similar stigma tools but had varying psychosocial results used different psychosocial construct tools and populations, thus producing contradictory results. Without more rigorous evaluation of these constructs, the cause and effect are not clear.

This review also reveals the need to create or adapt HIV stigma scales for cultural relevance and significance. In the systematic evaluation of the quality of the studies, the biggest challenge for content validity was how few studies included any modification for age or culture. Using consistent measures is an important goal for comparisons across cohorts, but the construct of HIV stigma may not be defined the same across cultures; utilizing a pre-existing scale in a new, different culture may neglect the measurement of culturally appropriate conceptions of the construct. Interestingly, the studies that utilized both qualitative and quantitative measures revealed ways in which the quantitative measures could fail to detect instances of stigma or beliefs about stigma that the participants found to be highly relevant on qualitative inquiry [28].

## Conclusions

This review provides evidence of the various HIV stigma measures being used to measure HIV stigma in paediatric populations. The available quantitative HIV stigma measures vary significantly, and few have been adapted for paediatric and adolescent populations or for the resource-limited settings in which most HIV-infected children live. Reliable, valid measures to quantify HIV stigma for populations living with HIV are critical to programmes’ ability to monitor and reduce the impact of stigma.
